# Do marginal investments made by NHS healthcare commissioners in the UK produce the outcomes they hope to achieve? Observational study

**DOI:** 10.1136/bmjopen-2015-009336

**Published:** 2015-11-06

**Authors:** Alicia O'Cathain, Fiona Sampson, Mark Strong, Mark Pickin, Elizabeth Goyder, Simon Dixon

**Affiliations:** School of Health and Related Research (ScHARR), University of Sheffield, Sheffield, UK

## Abstract

**Objective:**

To investigate the effect of targeted marginal annual investments by local healthcare commissioners on the outcomes they expected to achieve with these investments.

**Design:**

Controlled before and after study.

**Setting:**

152 commissioning organisations (primary care trusts) in England.

**Methods:**

National surveys of commissioning managers in 2009 and 2010 to identify: the largest marginal investments made in four key conditions/services (diabetes, coronary heart disease, chronic pulmonary airways disease and emergency and urgent care) in 2008/2009 and 2009/2010; the outcomes commissioners expected to achieve with these investments; and the processes commissioners used to develop these investments. Collation of routinely available data on outcomes commissioners expected from these investments over the period 2007/2008 to 2010/2011.

**Results:**

51% (77/152) of commissioners agreed to participate in the survey in 2009 and 60% (91/152) in 2010. Around half reported targeted marginal investments in each condition/service each year. Routine data on many of the outcomes they expected to achieve through these investments were not available. Also, commissioners expected some outcomes to be achieved beyond the time scale of our study. Therefore, only a limited number of outcomes of investments were tested. Outcomes included directly standardised emergency admission rates for the four conditions/services, and the percentage of patients with diabetes with glycated haemoglobin <7. There was no evidence that targeted marginal investments reduced emergency admission rates. There was evidence of an improvement in blood glucose management for diabetes for commissioners investing to improve diabetes care but this was compromised by a change in how the outcome was measured in different years. This investment was unlikely to be cost-effective.

**Conclusions:**

Commissioners made marginal investments in specific health conditions and services with the aim of improving a wide range of outcomes. There was little evidence of impact on the limited number of outcomes measured.

**Strengths and limitations of this study**

- Between 2006 and 2013, primary care trusts were expected to improve access to services, the quality of services, health outcomes and efficiency, and reduce health inequalities. Annual marginal investments in specific services or health conditions was one approach taken by local healthcare commissioners to improve outcomes.
- The strength of the study was that it aimed to address an evidence gap by investigating the effect of these targeted marginal annual investments on the outcomes commissioners expected to achieve with these investments.
- Surveys of healthcare commissioners identified investments made; routine data was used to test the impact on expected outcomes using a controlled before and after design.
- Limitations were the limited amount of routine data available nationally, the lack of consistent measurement of some indicators over time, and the difficulty attributing change to the investments made.

## Background

In some countries the responsibility for ensuring that healthcare is available to the population lies with commissioning organisations.^1^ Approaches to commissioning vary in terms of type of organisation, funding sources and terminology, both between countries and over time within the same country. Despite this, evidence relating to commissioning models in one country at a particular time may be informative for other contexts.

The term ‘commissioning’ is used in the National Health Service (NHS) in England to describe a wide range of activities such as assessing needs, allocating resources to best meet those needs, establishing contractual arrangements with service providers, and monitoring outcomes.^2^ It is distinguished from the purchasing of healthcare by its proactive and strategic intent.^3^ Commissioning healthcare in England occurs at different levels, from an individual perspective of using personal budgets through to a regional or national perspective of commissioning specialised hospital services.^4^ A large amount of commissioning occurs at a local level by groups acting on behalf of local populations (we will call this local commissioning within this paper). Between 2006 and 2013 local commissioning was undertaken by 152 primary care trusts (PCTs) in conjunction with practice-based commissioners from primary care. In April 2013 PCTs were replaced by Clinical Commissioning Groups (CCGs) and a national NHS commissioning board (more recently called NHS England). PCTs were responsible for commissioning healthcare to average populations of around 330 000 and were expected to achieve outcomes including improvements in the quality of services, more efficient use of resources (eg, reduction in emergency admission rates), improvements in access to services (eg, reductions in waiting times for outpatients), improvements in health, reductions in health inequalities, and financial balance.

### Evidence of the effectiveness of local commissioning

There is little research evidence of the effectiveness of local commissioning. The tendency has been to study processes and organisational issues due to the speed of change around introducing new local commissioning models.^5^ An overview of the impact of commissioning reforms in England identified different ways of measuring the outcomes of commissioning reforms.^3^ The conclusion of the review was that there is relatively little robust research evidence about the performance of local commissioning. The authors described evidence of a limited effect on shifting services out of hospital but cite achievement of reductions in waiting times for treatment, procuring new services and securing financial balance after a deficit. They found few studies which quantified impact but cite evidence of variation in service use around the country as an indication of continuing inefficiency. A comprehensive literature review of the impact of clinical commissioning models also concluded that research evidence is limited.^6^ This review identified some evidence of reductions in referrals from primary to secondary care, occupied bed days for emergency admissions, prescribing costs and waits for elective surgery and outpatients. Other studies have found that local commissioners who prioritised specific outcomes, such as numbers of people stopping smoking and breast screening coverage, achieved more improvement in these outcomes than those not prioritising them.^7^ A cross-sectional study showed that as expenditure by local commissioners increased, mortality decreased for circulatory diseases and cancer.^8^ However, this was a cross-sectional study and it is important to measure the effect of change over time in actions taken by local commissioners in order to better understand causal relationships.

### Addressing the evidence gap

Since there is limited evidence of the effectiveness of local healthcare commissioning, we aimed to contribute to the evidence base by focusing on the effect of marginal investments by local commissioners. Research has shown that commissioners are more likely to make marginal investments or disinvestments related to priority setting and rationing than engage in large strategic change.^9^ Healthcare commissioners make marginal investments each year which they hope will deliver specific outcomes in a specific time frame. Our aim was to test whether local commissioners achieved what they hoped in the time frame expected. Our focus was not to describe or make judgements about the mechanisms by which commissioners hoped to achieve their goals, but to measure whether commissioners achieved these goals. We were also interested in the commissioning processes they used to develop these investments because of the national focus on the quality of commissioning at the time of our research, for example, the inclusion of clinicians in commissioning decisions. Therefore, a secondary aim was to measure the effect of different commissioning processes on any outcomes achieved.

Before embarking on the study we were aware of a number of difficulties associated with using nationally available routine data to measure the effect of marginal investments by local commissioners. The first was that the analysis was dependent on the availability of routine data relevant to changes commissioners wished to effect, in particular that it needed to be measured consistently over a period of a number of years. It is also the case that the changes that commissioners attempt to achieve can be affected by many other groups such as acute and primary care providers, making attribution to local commissioners’ investments difficult. We undertook the study despite these difficulties because it is important to understand the extent to which commissioners are achieving their goals. To address these difficulties we used a controlled before and after design (comparing changes in commissioners making investments compared with those not making investments) over a short time period (to minimise the chances of changes being made to measurements collected routinely), with a large number of local commissioners (to minimise the effect of changes made by other organisations). We discuss these issues further in the strengths and limitations section of the paper, and suggest an alternative approach in the implications section of the paper.

## Methods

### Focus on marginal investments

As stated earlier, there are many aspects to commissioning.^2^ Our study focused on one aspect: healthcare commissioners making targeted marginal investments each year with the expectation that these will affect outcomes such as improving the health of a patient group or reducing unnecessary attendance at secondary care within an expected time frame. Such investments are distinct from unplanned changes to budgets, or budgetary increases for undefined purposes (eg, to take account of inflation). These marginal changes—which we call commissioning initiatives—may require financial investment, reconfiguration of services, or disinvestment to achieve desired outcomes. During the time period of our study, although some PCTs were in deficit, the context was one of increasing resource allocation to PCTs so PCTs were making marginal investments rather than disinvestments.

Commissioning is a complex process which attempts to produce multiple outcomes. We focused on specific conditions and services to allow us to explore the relationship between specific investments and specific outcomes. We selected three long-term conditions—diabetes, coronary heart disease (CHD) and chronic obstructive pulmonary disease (COPD)—and emergency and urgent care services because they collectively represent a major burden of potentially preventable chronic and acute ill-health at population level, and are major areas of both primary and secondary care spending.^8^

### Surveys to identify investments, expected outcomes and commissioning processes

We needed to identify from local commissioners the marginal investments they made in a particular financial year, the outcomes they hoped to achieve through these investments, the time frame in which they hoped to achieve these outcomes and the processes they used to commission initiatives using these investments. To do this, we undertook surveys of commissioning managers in PCTs, the details of which have been published elsewhere.^10^ Briefly, we undertook telephone surveys of commissioning managers in all 152 PCTs in 2009 and in 2010. Each year we undertook up to four structured telephone interviews in each participating PCT, selecting a commissioner leading each of our four conditions/service. We used a structured questionnaire to identify the largest initiative that had been commissioned by the PCT (ie, investment of new money or reconfiguration or disinvestment of services) and that had started in the previous financial year. In the 2009 survey we also asked if a larger initiative had occurred in the previous 2-year period because this could affect outcomes in the timeframe we were studying. Commissioners were then asked to describe: whether the initiative was a reconfiguration, investment or a disinvestment; the scope of the initiative (eg, whether it was aimed at all patients with diabetes in the PCT or a specific group of patients with diabetes); details of funding including the total amount of financial resource allocated; expected outcomes and when they were expected; and processes used to develop and manage the initiative. The processes included the extent to which key stakeholders such as clinicians or patients were involved in developing the initiative, the use of needs assessment and an evidence-based approach, the quality of management and leadership for the initiative and the extent of barriers to making changes associated with the initiative.^10^ We submitted the study to South Humber Research Ethics Committee reference 08/H1305/88 which classed it as a service evaluation.

### Routine data for measuring expected outcomes

Having identified the outcomes which commissioners wished to achieve with their marginal investments, we then looked for data to measure these outcomes for all PCTs over the time period 2007/2008 to 2010/2011. Outside the context of a prospective evaluation, measuring the outcomes achieved by local commissioners requires the availability of routine data. Most routine data at PCT level relate to the measurement of activity and few relate directly to health outcomes. Key sources of routine data relevant to the outcomes commissioners hoped to achieve with their investments were the Hospital Episode Statistics (HES) for measuring hospital utilisation rates and the Quality Outcomes Framework (QOF) for measuring quality in primary care. There was no routine data collected over the full-time period and of good enough quality to measure some outcomes expected by local commissioners: access to care, movement of service provision into the community or emergency department use. For example the quality of HES outpatient data and HES emergency department data was poor in the time period we addressed.

We located data on seven outcomes expected by commissioners: HES data on emergency admission rates for each of the four conditions/service, and QOF data on three disease-specific health outcomes in diabetes. The disease-specific outcomes were the proportion of patients with diabetes in a PCT with HbA1c<7, the proportion of patients with diabetes in a PCT with HbA1c<9, and the percentage of patients with diabetes undergoing retinopathy screening. These outcomes were relevant to only some of the investments. For example, commissioners expected some investments in diabetes to reduce emergency admissions and some to improve health. When survey respondents described the expected outcome for their diabetes investment as ‘improved disease-specific health outcome’, we assigned this initiative to a specific QOF indicator. We assigned any diabetes initiative designed to help patients manage their condition better to an expected outcome of an improvement in HbA1c. We assigned any initiative about retinopathy to the expected outcome of an increase in patients with diabetes undergoing retinal screening.

HES and QOF data were obtained for all years between 2007/2008 and 2010/2011, that is, 1 year prior to the marginal investments and at least 1 year after the marginal investments. From the HES data we calculated the annual directly age and sex standardised emergency admission rates per 100 000 population for each of our three conditions and for all emergency admissions using documented standardisation methods.^11^ We used the International Classification of Diseases 10th Edition (ICD 10) primary diagnosis codes E10-E14 for diabetes, J40-J44 for COPD and I2-I25 and I50 for CHD. We obtained QOF data from the NHS Information website for the period 2007/2008 to 2010/2011 (http://www.ic.nhs.uk/statistics-and-data-collections/audits-and-performance/the-quality-and-outcomes-framework)

### Analysis

We anticipated that, where a local commissioner had started a new initiative in 2008/2009 with specified primary outcomes expected within 2 years, we would be able to see a change in those expected outcomes compared with local commissioners reporting no investments in that year. We expected the size of change in outcome to be related to the size of marginal investment made per year per patient with the condition.

Separate analyses were undertaken for individual outcomes for individual conditions. We excluded the PCTs making reconfigurations and disinvestments (2 PCTs in 2008/2009 and 14 in 2009/2010) so that the analysis focused on investments only. We excluded PCTs which had their largest initiative in the previous 2 years to ensure that outcomes from initiatives in previous years did not contaminate the analysis. We compared outcomes in 2010/2011 (after investment) with outcomes in 2007/2008 (before investment) for any initiatives with outcomes expected within 2 years for 2008/2009 initiatives and within 1 year for 2009/2010 initiatives. Where a PCT had invested in 2008/2009 and 2009/2010, both investments were included.

We undertook a linear regression with the outcome in 2010/2011 as the dependent variable, adjusting for the outcome in 2007/2008. We then tested the size of investment per patient with the condition as a continuous variable, with PCTs that had made no investment included as zero. We adjusted for the potential confounding variables of deprivation, age structure of population (% PCT population aged over 75) and the proportion of single-handed practices within a PCT because these have been found to be associated with our selected outcomes.^12^ ^13^ We undertook weighted least squares regression weighted by the denominator of rates used in the dependent variable to account for PCT population size.

### Cost-effectiveness modelling

We considered the cost-effectiveness of effective investments only—HbA1c for patients with diabetes. We used an existing model to estimate the cost per QALY of investments aimed at reducing HbA1c.^14^ The health benefit—the incremental quality adjusted life years—was estimated by using risk equations to determine health outcomes based on changes in HbA1c. Cost-effectiveness estimates for investments were then obtained by dividing the incremental costs of PCT investments (net of any savings associated with better health outcomes) by the incremental quality adjusted life years. The model used addresses type 2 diabetes, although our initiatives encompassed type 1 as well as type 2 diabetes. However, type 2 diabetes accounts for 90% of patients with diabetes in the UK.^15^

## Results

### Response rates to surveys

Fifty-one per cent (77/152) of PCTs agreed to participate in the survey in 2009 and a further 14 PCTs agreed to participate in 2010 (60%, 91/152).

### Marginal investments

Most of the largest commissioning initiatives started in 2008/2009 and 2009/2010 rather than in earlier years (table 1). Commissioners described a wide range of size of investments (mean investment of £391k in 2008/2009 and mean investment of £362k in 2009/2010). The size and source of investments are reported in table 2 for each condition/service. In 2008/2009 two PCTs reported cost-neutral service reconfigurations and no PCTs reported disinvestments. In 2009/2010 there were 12 cost-neutral reconfigurations and two disinvestments reported.

**Table 1:** Numbers of largest initiatives starting each year

Year started	Diabetes	COPD	CHD	Emergency and urgent care
2006/2007	9	12	8	7
2007/2008	13	12	12	12
2008/2009	39	39	37	42
2009/2010	28	31	35	39

CHD, coronary heart disease; COPD, chronic obstructive pulmonary disease.

**Table 2:** Size of investment in largest initiatives in 2008/2009

	Diabetes N=39	COPD N=39	CHD N=37	Emergency and urgent care N=42
Investment (£)				
Mean	276 597	179 901	455 642	642 550
Median	200 000	100 000	255 000	440 000
Range	10–1500 k	6.5–800 k	0–7198k	0–4000k
Mean investment per patient with condition (£)	17.88	16.61	27.63	2.15
Type of investment %recurrent	85% (33/39)	76% (28/37)	86% (31/36)	68% (27/40)
Source of budget standard PCT	92% (36/39)	84% (32/38)	83% (30/36)	95% (39/41)

CHD, coronary heart disease; COPD, chronic obstructive pulmonary disease.

### Outcomes expected

We asked respondents for one or two main or primary outcomes they expected to achieve for their largest marginal investment. We also asked for secondary outcomes and when they would be likely to see any change in outcomes (immediately, within 1 year, within 2 years, longer than 2 years). The outcomes which commissioners expected to achieve were very similar for investments starting in 2008/2009 and 2009/2010. The most common primary outcomes expected were a reduction in emergency admissions (48% of initiatives) and improved disease-specific health outcomes (40% of initiatives). The most common main outcomes which commissioners expected to occur immediately or within 1 year were a reduction in emergency admissions (36%), improved disease-specific outcomes (23%), increase in access to care (20%), movement of care into the community (20%) and ‘other’ (18%). As stated in the methods, we were able to find routine data for emergency admissions and diabetes-specific health outcomes only. For diabetes, nine PCTs invested in initiatives which they expected to reduce emergency admissions, 23 invested in initiatives which they expected to improve disease-specific outcomes and 6 invested in initiatives which they expected to increase retinal screening.

### Outcomes achieved

Changes in outcome measures over time are displayed in table 3. The size of marginal investment did not predict the outcome for six of the seven outcomes measured (table 4). The percentage of patients with diabetes in a PCT with good blood glucose control was higher in PCTs which invested more to achieve this outcome, adjusting for baseline. There was a half percent increase in the % PCT patients with diabetes with HbA1c<7 for every extra £10 invested per year per diabetes patient (p=0.023). However, this finding was compromised by a change in the way the indicator was measured in QOF in different years. A threshold of HbA1c<7.5 was applied in QOF in 2007/2008 and 2008/2009; this was changed to HbA1c<7 in 2009/2010 and 2010/2011.

**Table 3:** Change in outcomes for PCTs with large, small and no investments

Size of investment	Number of PCTs with investment	Outcome in 2007/2008	Outcome in 2010/2011	Change in outcome
Diabetes emergency admissions per 100 000				
Large	9	94	103	+9
Small	–	–	–	–
None	12	82	89	+7
COPD emergency admissions per 100 000				
Large	25	175	186	+11
Small	18	169	184	+15
None	10	167	198	+31
CHD emergency admissions per 100 000				
Large	12	343	291	−52
Small	5	272	238	−34
None	10	291	256	−35
All emergency admissions per 100 000				
Large	17	9027	9653	+626
Small	12	8764	9199	+435
None	7	9109	9718	+609
Percentage of diabetes HbA1c<7				
Large	14	61	49	−12
Small	9	60	47	−13
None	12	60	46	−14
Percentage of diabetes HbA1c<9				
Large	14	87	82	−5
Small	9	88	82	−6
None	12	88	82	−6
Per cent undergoing retinopathy screening				
Large	4	80	83	+3
Small	2	82	86	+4
None	12	85	86	+1

CHD, coronary heart disease; COPD, chronic obstructive pulmonary disease; HbA1c, glycated haemoglobin; PCT, primary care trust.

**Table 4:** Effect of targeted marginal investment of £10 per patient with condition on expected outcomes

	Number of PCTs with investment	Number of PCTs without investment	Unadjusted β-coefficient (95% CI)	p Value	Adjusted β-coefficient (95% CI)	p Value	R^2^
Diabetes emergency admissions	9	12	−0.25 (−1.82 to 1.32)	0.761	0.07 (−1.69 to 1.83)	0.933	0.80
COPD emergency admissions	33	10	−1.84 (−4.84 to 4.80)	0.225	−1.69 (−4.56 to 1.19)	0.244	0.89
CHD emergency admissions	17	10	1.7 (−3.7 to 7.1)	0.532	3.2 (−2–2 to 8.6)	0.247	0.81
All emergency admissions	29	7	32 (−492 to 556)	0.902	26 (−547 to 601)	0.926	0.88
Diabetes HbA1c<7*	23	12	0.46 (0.06 to 0.86)	0.032	0.50 (0.1 to 0.9)	0.023	0.46
Diabetes HbA1c<9†	23	12	0.1 (−0.04 to 0.24)	0.094	0.1 (−0.04 to 0.24)	0.148	0.57
Per cent undergoing retinopathy screening	6	12	−1.0 (−2.7 to 0.7)	0.251	0.1 (−2.4 to 2.7)	0.911	0.45

*Measure changed over time from <7.5 in 2007/2008 and 2008/2009 to <7 in 2009/2010 and 2010/2011.

†Measure changed over time from <10 in 2007/2008 and 2008/2009 to <9 in 2009/2010 and 2010/2011.

CHD, coronary heart disease; COPD, chronic obstructive pulmonary disease; HbA1c, glycated haemoglobin; PCT, primary care trust.

### Effect of commissioning processes on outcomes

We tested process variables to see if the blood glucose outcome achieved was dependent on how an investment was developed and managed. There was no evidence that the outcome was dependent on whether the initiative was reported to have been instigated by the PCT, practice-based commissioners or a group of commissioners (p=0.659); whether specialist clinicians were involved in developing and shaping the initiative (p=0.834); whether any general practitioner was involved in developing and shaping the initiative (p=0.263); whether public health specialists were involved in developing and shaping the initiative (p=0.176); whether patients or the public developed and shaped the initiative (p=0.884); the extent to which there had been assessment of need, cost and evidence (p=0.792); the level of leadership and management (p=0.804); or the extent of lack of barriers to the initiatives relating to disinvestment and competing priorities (p=0.813).

### Cost-effectiveness

In order to evaluate the cost-effectiveness of any investments expected to improve blood glucose control, it was necessary to estimate the difference in the change in HbA1c between the investing and non-investing PCTs. We did not know the nature of the change in distribution of HbA1c, that is, whether the change in proportions was due to a small number of individuals crossing the lower threshold, or a more overall shift in the distribution. We assumed a lognormal distribution for HbA1c in the population of people with diabetes, and fitted the distribution to the data in table 5, to obtain summary statistics for use in our model. The investing and non-investing PCTs had a slightly different baseline distribution of HbA1c scores: 61% versus 60%. We took measures to adjust the estimates for the differences at baseline. We sampled values for the change in the non-investing group from the log-normal distribution, and applied the change to sampled values from the baseline investing group. This provided an estimate of what the 2009/2010 HbA1c scores would be for the investing group if they had not invested. The absolute difference in the mean HbA1c for the investing versus non-investing PCTs was small: 0.0065% lower for the investing PCTs. The aggregate discounted cost of the investment was £124 per patient. The lifetime net costs from the impact of investment on medication use and treating diabetes-related complications was a saving of £3. The lifetime gain in Quality-Adjusted life Years (QALYs) was 0.00 038, giving a cost-effectiveness ratio of £321 000 which is highly unfavourable against the conventional willingness-to-pay threshold of £20 000 per QALY. There was uncertainty around parameters in the economic model and probabilistic sensitivity analysis indicated a 15% chance that the investment was cost-effective.

**Table 5:** Change in distribution of HbA1c in PCTs making marginal investments versus those not

	Good control, n (%)	Moderate control, n (%)	Poor control, n (%)
PCTs that invested N=23			
2007/2008 (before)	≤7.5 (61%)	7.6–10 (27%)	>10 (12%)
2010/2011 (after)	≤7 (48%)	7.1–9 (34%)	>9 (18%)
PCTs that did not invest N=12			
2007/2008 (before)	≤7.5 (60%)	7.6–10 (28%)	>10 (12%)
2010/2011 (after)	≤7 (46%)	7.1–9 (36%)	>9 (18%)

HbA1c, glycated haemoglobin; PCT, primary care trust.

## Discussion

### Summary of findings

Each year healthcare commissioners make targeted marginal investments, expecting to achieve a variety of outcomes in the short and longer term. We found little data available nationally that allowed us to measure the outcomes which commissioners hoped to achieve with their marginal investments. The most robust outcome measure we found was emergency admissions for different conditions calculated using Hospital Episode Statistics data. We found no evidence of a reduction in emergency admissions associated with investments in our study. Some sources of routine data such as the Quality Outcomes Framework were not as useful as Hospital Episode Statistics because changes made to the indicators in different years made comparisons over time difficult. We found evidence of an improvement in good HbA1c control for diabetes related to the size of marginal investment, although this finding was compromised by a change in how the outcome was measured over time. In a cost-effectiveness model this impact was unlikely to be cost-effective.

### Putting findings in the context of other research

As described earlier in the paper, there is limited evidence about the effect of local commissioning on outcomes, particularly health outcomes.^3^ ^6^ A review of clinical commissioning found no studies which attempted to measure the effect of commissioning on clinical outcomes such as blood glucose levels in diabetes. Therefore, our study is unique in that aspect. There is evidence that one model of local commissioning—Total Purchasing Pilots in 1996/1997—reduced emergency admission bed days 16 which appears to conflict with our finding that there was no effect of local commissioning investments made by PCTs on emergency admission rates. The different results might be explained by the focus on admission rates in our study rather than bed days (which also includes length of stay), although it is interesting to note that a minority of total purchasing pilots had a sustained priority of impacting on emergency admissions, with some abandoning it as a priority possibly due to it being difficult to achieve.^16^ A systematic review showed that a large number of initiatives aimed at reducing emergency admissions have a limited research evidence base or have been shown not to work.^17^ That is, PCT commissioners in our study were trying to address a problem where little has been shown to be effective even under research conditions.

There is evidence that commissioning models have impacted on outcomes which we did not measure in our study. These include referrals to secondary care, prescribing costs, waiting times for elective treatment and outpatients,^16^ smoking cessation and breast screening coverage.^7^ There is also evidence that commissioning did not affect outcomes which we did not measure in our study.^16^ For example, PCTs with referral management schemes did not impact on outpatient attendance rates compared with PCTs without them.^18^ An additional complexity faced by local commissioners is that the types of outcomes expected of commissioning include potentially conflicting priorities. For example, local commissioners may need to trade off improved efficiency for improved access or equity.^19^

### Strengths and limitations

The main strengths of the study were that we attempted to measure the outcomes of local commissioning to address an evidence gap, that we focused on outcomes which commissioners themselves hoped to achieve in a large number of commissioning organisations, and that we used a controlled before and after design to measure change over time compared with PCTs making no investment. A related design has been used successfully in other studies to identify the effect of PCT initiatives on outpatient attendance rates.^18^ There were seven limitations. First, the data we collected on the investments may not have fully reflected reality because the initiatives invested in were often multicomponent, the level of detail we required was sometimes not known, or the detail was not known by the individual commissioners because they had not been in post for long. Second, we found very little routine data of good quality, which was measured consistently over the time period required, to allow us to measure the many outcomes expected by commissioners. For example, some of our commissioners invested in education and self-management training for type 2 diabetes because it can affect weight loss and smoking cessation^20^ but there was no national data at PCT level for these outcomes. Therefore, our study could underestimate the impact of commissioning on expected outcomes because it did not include measurement of some important outcomes. Third, changes in how the QOF indicators were measured over time limited their value to our study. Fourth, the analysis was undertaken at the PCT level with a small number of PCTs within each analysis resulting in low statistical power to detect differences, particularly if differences were small. Fifth, our analysis focused on single outcomes, requiring a large effect on a single outcome. Some effects may be small but still of significance. Also, rather than having a large effect on a single outcome, the initiatives PCTs invested in may have had a small effect on multiple outcomes.^14^ Indeed some outcomes further down the pathway of outcomes may be the most economically beneficial, for example, diabetes education programmes reduce smoking rates which cause longer term reductions in cardiovascular risk.^14^ Sixth, selection bias was likely to be present in our study. Only 60% of PCTs took part in the survey. These PCTs were not representative of all PCTs. They were less likely to be in financial deficit and more likely to have a good assessment during national reviews of the quality of commissioning.^10^ They also had higher emergency admission rates than non-responding PCTs although their disease-specific outcomes were identical. That is, they were PCTs in a position to invest, and wanting to invest to impact on a specific outcome because their commissioners perceived they had a problem relative to other PCTs. Finally, there is the limitation of attributing changes found to the investments made. Although we measured whether commissioners obtained the outcomes they expected, regardless of whether the intervention they invested in could deliver such an outcome, we did not consider the plausibility of these interventions impacting on the expected outcomes. This concern about attribution particularly applies to the outcomes measured using QOF because QOF is an incentive for general practices to improve health and healthcare and we did not control for any actions taken by general practices. Researchers undertaking similar types of studies have faced this challenge of attribution when using QOF.^21^

### Implications

We detected little or no change in outcomes associated with marginal investments made by local commissioners. There are three possible explanations for this finding. First, that the investments did not produce improved outcomes. It may be unfair to draw this conclusion because of some of the limitations of our study. However, it is worth noting that we could find relatively few studies which did identify commissioning affecting the outcomes we measured. It is also the case that local commissioners may be trying to affect some outcomes that are very important but which are difficult to improve. In particular, a review of 1530 controlled studies evaluating interventions to reduce emergency admissions concluded there was insufficient or no convincing evidence to support most of them.^17^ A second possible explanation is that outcomes were produced and were potentially detectable but were not detected due to limitations in the available data. This is a likely conclusion to draw from our study because we only measured a limited number of outcomes due to the lack of availability of good quality routine data. A third possible explanation is that outcomes were produced but were not detectable due to the changes being small in multiple conflicting outcomes occurring in a constantly changing complex environment. The complexity of the pathways by which any investments are expected to have an impact on patient outcomes or service activity levels, and the likely interaction with many confounding factors which also influence these outcomes, means that even if an investment has a relatively clear evidence base it is not possible to assume that the impact will be similar when replicated in a different setting or different population. Additionally, some outcomes may be in conflict as commissioners may need to trade off improved efficiency for improved access or equity.^19^

In terms of transferability of these findings, we undertook our study at a time when many local commissioners were receiving increasing resources and were able to invest in new initiatives. Our commissioners described few examples of disinvestments. Local commissioners may be more focused on disinvestment in times of economic downturn. Additionally, local commissioning models are different in other countries and have already changed in England since we completed this study. Even so, measuring the effect of local commissioning on outcomes is an important issue and measuring the effect of marginal investments remains relevant given the continuing focus of new commissioning models in England on marginal changes.^22^ Data availability, and attribution of effect, are likely to be the largest challenges facing those wishing to measure the effect of local commissioning. Regardless of these challenges, it remains important to understand the impact of local commissioning. We would recommend that, rather than take the approach we took here, future evaluations are built into the commissioning process so that there is a clear specification of anticipated outcomes that will allow appropriate outcome data in the target areas (and appropriate control groups) to be gathered. Data availability for some key outcomes was poor for our national study but more data are available locally than nationally, and available at general practice level, that are helpful for modelling the effect of local commissioning decisions.^23^ The challenge of attribution can be addressed by gathering detailed information about the range of new initiatives occurring in each local commissioning area in addition to those funded by the local commissioners.

## Conclusions

Local commissioners made investments with the aim of improving a wide range of outcomes. There is limited consistently measured routine data relevant to these outcomes, which is available at a national level. We found little or no impact on the limited number of outcomes we could measure—reduction in emergency admissions for diabetes, CHD, COPD and all conditions; and blood glucose management for diabetes.
